# Enhancement of Strength–Ductility Synergy of Al-Li Cast Alloy via New Forming Processes and Sc Addition

**DOI:** 10.3390/ma17071558

**Published:** 2024-03-28

**Authors:** Shulin Lü, Zhaoxiang Yan, Yu Pan, Jianyu Li, Shusen Wu, Wei Guo

**Affiliations:** State Key Lab of Materials Processing and Die & Mould Technology, School of Materials Science and Engineering, Huazhong University of Science and Technology, Wuhan 430074, China; shulin317@hust.edu.cn (S.L.); m202271082@hust.edu.cn (Z.Y.);

**Keywords:** Al-Li cast alloy, squeeze casting, ultrasonic treatment, Sc addition, microstructure, mechanical properties

## Abstract

In this study, concurrent enhancements in both strength and ductility of the Al-2Li-2Cu-0.5Mg-0.2Zr cast alloy (hereafter referred to as Al-Li) were achieved through an optimized forming process comprising ultrasonic treatment followed by squeeze casting, coupled with the incorporation of Sc. Initially, the variations in the microstructure and mechanical properties of the Sc-free Al-Li cast alloy (i.e., alloy A) during various forming processes were investigated. The results revealed that the grain size in the UT+SC (ultrasonic treatment + squeeze casting) alloy was reduced by 76.3% and 57.7%, respectively, compared to those of the GC (gravity casting) or SC alloys. Additionally, significant improvements were observed in its compositional segregation and porosity reduction. After UT+SC, the ultimate tensile strength (UTS), yield strength (YS), and elongation reached 235 MPa, 135 MPa, and 15%, respectively, which were 113.6%, 28.6%, and 1150% higher than those of the GC alloy. Subsequently, the Al-Li cast alloy containing 0.2 wt.% Sc (referred to as alloy B) exhibited even finer grains under the UT+SC process, resulting in simultaneous enhancements in its UTS, YS, and elongation. Interestingly, the product of ultimate tensile strength and elongation (i.e., UTS × EL) for both alloys reached 36 GPa•% and 42 GPa•%, respectively, which is much higher than that of other Al-Li cast alloys reported in the available literature.

## 1. Introduction

Aluminum–lithium (Al-Li) alloys have received widespread attention in the aerospace field owing to their exceptional characteristics, such as their high elastic modulus and low density [1,2]. It has been indicated through research that for every 1 wt.% of Li incorporated into the Al-Li alloy, the density decreases by 3%, while the elastic modulus increases by 5–6% [3,4]. Current research on Al-Li alloys mainly concentrates on third- or fourth-generation Al-Cu-Li alloys (with Li content as low as 0.6 wt.%), which are commercially employed in components of relatively simple structures, such as wings and fuel storage tanks [5,6,7]. However, the production of existing Al-Cu-Li alloys typically involves plastic deformation techniques, such as hot extrusion and rolling, to make sheets or bars (i.e., wrought Al-Li alloys) [8,9]. Due to the limitation of the plastic forming process, some large and complex parts can only be formed via the casting method. Moreover, Al-Li cast alloys are isotropic, which allows for a higher Li content to enhance their potential for reduced weight and increased elastic modulus [10]. Therefore, it is important to develop Al-Li cast alloy materials or castings.

However, poor toughness is an important technical bottleneck that restricts the development of Al-Li cast alloys. Typically, the elongation of currently available heat-treated Al-Li cast alloys does not exceed 4%, and the as-cast elongation is even less than 2%. For heat-treatable alloys, the final properties in the heat-treated state are jointly influenced by the heat treatment process, as well as the solidification microstructure and properties of the as-cast alloy. Micro-alloying is one of the common means of improving the solidification microstructure and properties of the Al-Li alloy. Among many microaddition elements, Sc is the most effective in refining the solidification of the microstructure and strengthening the Al-Li cast alloy [11,12,13,14]. Wang et al. [14] reported that the addition of 0.1 wt.% Sc to the Al-2Li-3Cu-0.1Zr cast alloy led to grain refinement and an increase in as-cast UTS from 168 MPa to 242 MPa, with the elongation being elevated from 6.6% to 8.6%.

In addition to microalloying, the preparation process is another important factor that affects the solidification microstructure and properties of the alloy. For Al-Li cast alloys, the production of Al-Li casting frequently employs non-vacuum melting combined with inert gas shielding and a covering agent, followed by gravity casting due to the process’s simplicity and economic efficiency [14,15,16]. For instance, Chao et al. [16] developed an Al-2.5Li-5Mg-1Cu alloy through non-vacuum melting plus gravity casting, achieving an as-cast UTS of 253.5 MPa and an elongation of 1.01%. Owing to the “genetic effect”, the elongation of the alloy was still only 3.43% after T6 heat treatment. Therefore, in order to meet the demand for lightweight and high-performance castings in aerospace structural components, it is urgent to develop new preparation and forming technologies suitable for high-strength and ductile Al-Li cast alloys.

Squeeze casting can apply high pressure to metal melts, which facilitates the solidification process, serving as a reliable technique for producing high-quality Al-Li cast alloys. High-pressure solidification confers advantages that cannot be achieved through gravity casting, including rapid cooling, oxidation prevention, and the refinement of grain and second phase, as well as the reduction or elimination of porosity [17,18]. Fan et al. found that the UTS and elongation of the squeeze-cast Al-2.47Li-1.49Cu alloy were 221 MPa and 2.9%, which were 13.3% and 262.5% higher than those of the Al-2.47Li-1.49Cu alloy prepared via gravity casting, respectively [18]. However, there is still a problem of composition segregation in squeeze-cast Al-Li alloys, such that their mechanical properties are not satisfactory. As we all know, ultrasonic treatment (UT) of the melt is an environmentally friendly technology that not only aids in grain refinement and homogenization of the microstructure but also enhances the efficiency of degassing and refining process [19]. Therefore, the application of UT technology to Al-Li melt preparation is expected to eliminate compositional segregation and reduce gas in the melt significantly. However, there is a lack of systematic research on the microstructure evolution and mechanical properties of Al-Li alloys formed via squeeze casting assisted by ultrasonic treatment.

In this study, the effects of forming processes (i.e., GC, SC, and UT+SC) and Sc addition on the microstructure and mechanical properties of an Al-2Li-2Cu-0.5Mg-0.2Zr cast alloy were investigated. The mechanisms for microstructure evolution and strengthening and toughening are also discussed.

## 2. Materials and Methods

**Figure 1:**
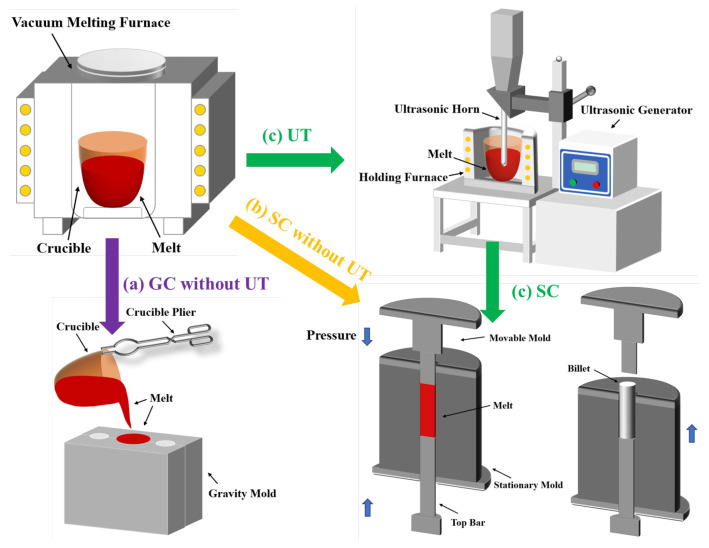
Schematic diagram of three preparation processes for Al-Li cast alloys, including melting, ultrasonic treatment, and casting.

The actual chemical compositions of Al-2Li-2Cu-0.5Mg-0.2Zr-(0.2Sc) alloys were determined using the inductively coupled plasma (ICP) technique, presented in Table 1. The raw materials for the two Al-Li alloys consist of pure Al, Mg, and Cu ingots, as well as Al-10Li, Al-10Zr, and Al-2Sc master alloys, which were added into the vacuum melting furnace for melting. During the melting process, the vacuum degree of the melting furnace was kept at −0.1 MPa~−0.08 MPa. Three different forming processes for Al-Li cast alloys are shown in Figure 1: (a) GC (gravity casting) without UT (ultrasonic treatment); (b) SC (squeeze casting) without UT; (c) SC assisted with UT. Taking UT+SC as an example, the preparation process is as follows: After melting the raw materials, UT is applied for refining. The UT process is carried out at a temperature range of 660–680 °C for 2 min under the protection of high-purity argon gas. It is worth noting that the same ultrasonic temperature and time are used for all specimens in this study. The treated melt is then poured into a metal mold, preheated to a temperature of 200 °C. Finally, the moveable mold is rapidly compressed and maintained at 50 MPa for 15~20 s until the melt is completely solidified, obtaining an ingot with dimensions of φ 30 × 90 mm.

Samples taken from different alloys at the same location were ground, polished, and etched for microstructure observation using the GeminiSEM300 field emission SEM. Powder samples taken from different alloys were scanned in the range of 20° to 90° at a rate of 10°/min using an XRD-7000 X-ray diffractometer to determine the phase composition. For the preparation of electron back-scattered diffraction (EBSD) samples, mechanical grinding was performed using abrasive and silica suspension, followed by precise ion etching using the Gatan PECS II 685 instrument for 30~40 min at a voltage of 2~4 keV. Then, EBSD observation was carried out using the GeminiSEM300 field emission SEM with an Aztec Nordlys Max3 probe, and step lengths were selected at 2~5 μm according to various average grain sizes. EBSD data were analyzed using AztecCrystal2.1.259 software. Electron probe microanalysis (EMPA) was performed using an EPMA-8050G to analyze the elements qualitatively or quantitatively in the micro-regions on the sample surface. Unlike SEM samples, EPMA samples do not need to be etched with Keller’s corrosive before characterization. To conform with the GB/T228.1-2010 Chinese Standard (equivalent to ASTM A370-2016) [10], tensile samples were obtained from the ingot through a process of wire cutting and machining. To guarantee accuracy, three tensile rods were taken from each sample, and the average values were taken as the final mechanical properties.

## 3. Results

### 3.1. Microstructure Evolution of Al-Li Cast Alloy under Different Forming Processes

**Figure 2:**
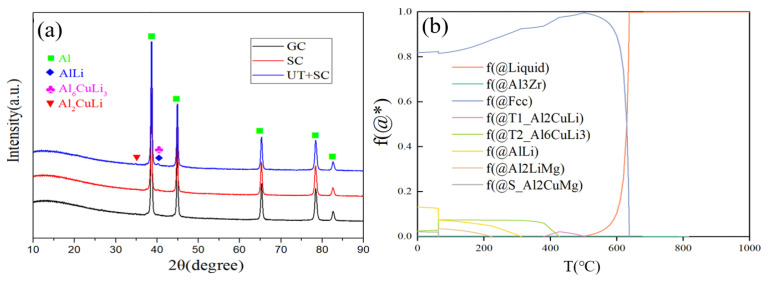
(**a**) XRD patterns of Al-Li cast alloy without Sc addition (i.e., alloy A). (**b**) Solid-phase volume fraction–temperature curve of as-cast alloy A under equilibrium solidification condition.

The XRD patterns of the Al-Li alloys reveal the existence of α-Al, AlLi, Al_6_CuLi_3_, and Al_2_CuLi phases, as shown in Figure 2a. The phase type and diffraction peak intensity in the UT+SC alloy show no substantial alteration in comparison to GC and SC alloys. Therefore, the UT and SC processes seem to have no impact on the phase composition of the Al-Li alloy. Figure 2b shows the solidification curves (i.e., phase diagram) of the as-cast Al-Li alloy under equilibrium solidification condition, which is simulated using the Pandat2022 software. It can be seen that the liquid metal starts to solidify from about 650 °C and is complete at about 500 °C, resulting in a final α-Al phase content of approximately 82%. The phase diagram shows that there is less than 1.8% Al_6_CuLi_3_ or Al_2_CuLi phase in the alloy during the equilibrium solidification. However, according to the XRD pattern of the alloy, the diffraction peak of the Al_6_CuLi_3_ and Al_2_CuLi phases can hardly be detected. This discrepancy may be attributed to the unavoidable elemental burnout and non-equilibrium solidification behavior during the smelting and squeeze casting process, resulting in some discrepancies between the simulated and experimental results. Overall, the simulation results are in general agreement with the experimental results, except for trace amounts of the second phase that are difficult to detect.

**Figure 3:**
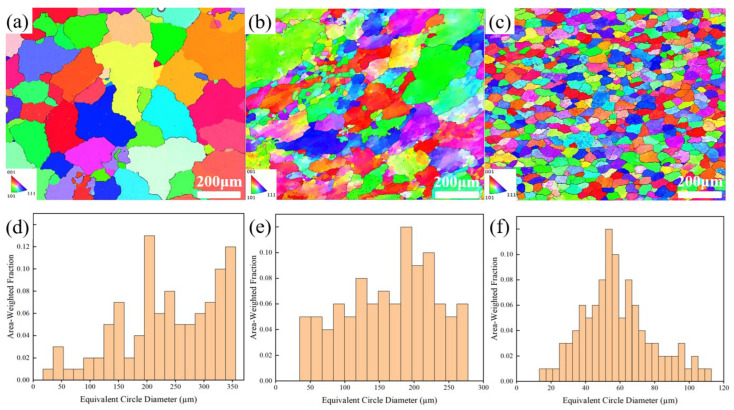
Inverse pole figure (IPF) and grain size distribution of Al-Li cast alloy without Sc addition (i.e., alloy A) prepared via (**a**,**d**) GC, (**b**,**e**) SC, and (**c**,**f**) UT+SC.

Figure 3 exhibits the grain size distribution and inverse pole figure (IPF) of three Al-Li alloys under different forming processes, including GC, SC, and UT+SC. It is evident that forming processes play a crucial role in determining the morphology and controlling the size of α-Al grains. The comparative analysis between Figure 3a,b indicates that the GC alloy has coarser grains while the SC alloy exhibits a considerably finer grain size. In particular, the UT+SC alloy has the smallest grain size, with a more uniform distribution and enhanced roundness, as shown in Figure 3c. Based on the statistical analysis of the grain size distribution data presented in Figure 3d–f, it was found that the average grain size of the SC alloy decreased from 186 μm to 104 μm compared to the GC alloy, representing a significant reduction of 44%. However, coarse grains (which can be as large as 250 μm or more) are still present in SC alloys, accompanied by a broad variation in grain size. After UT+SC, the average grain size decreased to 44 μm, which was 76% and 58% less than that of the GC and SC alloys, respectively.

**Figure 4:**
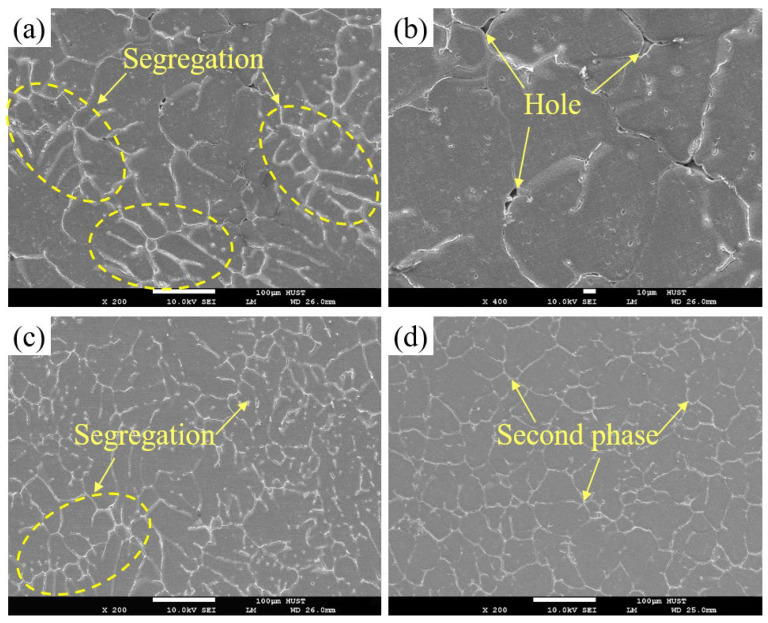
SEM images of Al-Li cast alloy without Sc addition (i.e., alloy A) prepared via (**a**,**b**) GC, (**c**) SC, and (**d**) UT+SC.

To further investigate the influence of the forming process on the microstructure evolution, the SEM images of alloy A produced via GC, SC, and UT+SC are shown in Figure 4. In the case of GC alloy, the second phase is not only coarse in size and non-uniformly distributed (i.e., compositional segregation, which is marked by yellow ellipse); there are also some large holes (Figure 4a,b). After SC, the second phase undergoes significant refinement and the pores are disappeared, but local compositional segregation is still present (Figure 4c). As shown in Figure 4d, the UT+SC process can effectively eliminate segregation and achieve the complete disappearance of pores, while reducing the size difference of the second phase and making the distribution of these phases more uniform.

**Figure 5:**
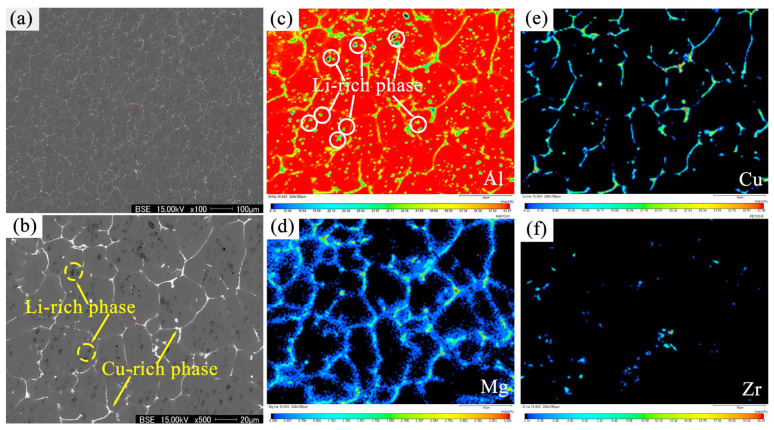
BSE images (**a**,**b**) and the main elemental mapping distribution of the Al-Li alloy: (**c**) Al; (**d**) Mg; (**e**) Cu; (**f**) Zr.

Figure 5 shows the BSE images and EPMA (electron probe micro-analyzer) elemental maps of alloy A prepared via UT+SC, illustrating the distribution of Al, Cu, Mg, and Zr elements. Based on the imaging principle of BSE, the higher the atomic number of the intermetallic element, the brighter the intermetallic layer. Except for gray α-Al grains, there are a large number of bright and dark second phases. The bright second phases are predominantly located at the grain boundaries, forming a reticulated structure, as shown in Figure 5a,b. For the EPMA, the brighter the color, the greater the concentration of a particular element in the region. It is evident that the bright second phase is a Cu-rich phase, some of which has a small amount of Mg elemental enrichment (i.e., speculating that it may be an Al_2_CuMg phase). However, it is difficult to speculate on the type of these Cu-rich phases, such as Al_6_CuLi_3_ and Al_2_LiMg phases, beyond being able to determine the Cu-containing elements. This is because the atomic number of Li element is too low to be detected by the EPMA. However, the presence of some Li-rich phases can also be indirectly confirmed by combining Figure 5a–c,f. Obviously, there are some instances of Al-poor phases in Figure 5c (i.e., the black second phases shown in Figure 5b), and there are no other elements, such as Cu, Zr, or Mg, overlapping with them (Figure 5d,f). Therefore, it can be assumed that the second black phase is a Li-rich phase (i.e., AlLi phase). This finding provides additional evidence for the existence of Li-rich and Cu-rich phases, which is in better agreement with the XRD pattern and simulations results from the Pandat2022 software.

### 3.2. Mechanical Properties of Al-Li Cast Alloy under Different Forming Processes

**Figure 6:**
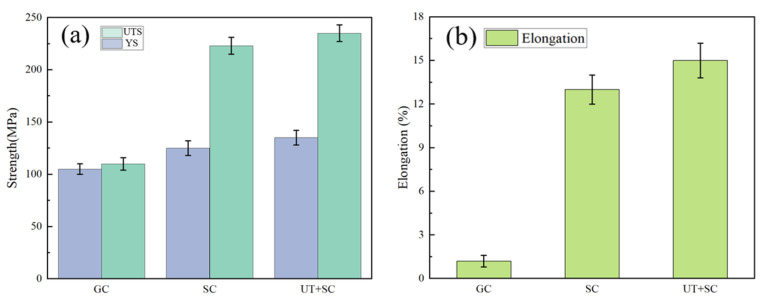
Mechanical properties of Al-Li alloys under different processes: (**a**) strength; (**b**) elongation.

Figure 6 shows the as-cast mechanical properties of the Al-Li alloy prepared via three different processes, including GC without UT, SC without UT, and UT+SC. Obviously, the forming processes have a great influence on the as-cast strength and ductility of the Al-Li cast alloy. For the GC alloy, the UTS, YS, and elongation are 110 MPa, 105 Mpa, and 1.2%, respectively. After SC, the UTS, YS, and elongation of the Al-Li alloy are 220 MPa, 125 Mpa, and 13%, which are 100.0%, 19%, and 983% higher than those of the GC alloy, respectively. When UT was combined with the SC process, the Al-Li alloy had the best comprehensive as-cast mechanical properties. The UT+SC alloy’s UTS, YS, and elongation are 235 MPa, 135 Mpa, and 15%, which have been increased by 6.8%, 8%, and 15.4% compared to the SC alloys. Meanwhile, its UTS, YS, and elongation are 113.6%, 28.6%, and 1150% higher than those of the GC alloy, respectively.

**Figure 7:**
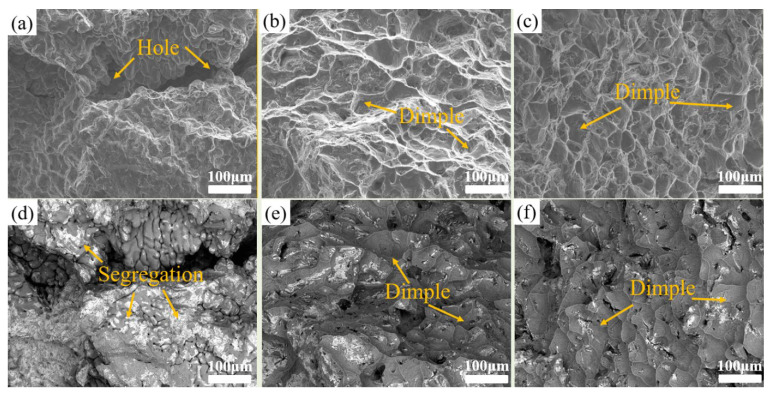
Fracture surface morphology along the cross section of tensile specimens cut from various Al-Li alloys prepared via different methods: (**a**,**d**) GC; (**b**,**e**) SC; (**c**,**f**) UT+SC.

In order to gain an insight into the mechanical properties of Al-Li cast alloys during various forming processes, the fracture morphology along the cross section for tensile specimens was observed by SEM and BSE, as shown in Figure 7. In general, toughness fracture is generally dependent on the size, distribution, and number of dimples. The more uniform the distribution and deeper the size of dimples, the higher the plasticity of the alloy. For the GC alloy, the fracture surface exhibits large holes without dimples, indicating characteristics of brittle fracture. Additionally, a substantial amount of bright secondary phases (i.e., Cu-rich phases) are presented on the surface, thereby confirming the existence of severe compositional segregation in the GC alloy (Figure 7a,d). After SC, the fracture morphology changed significantly. Not only are the large holes basically eliminated, a large number of small dimples also appear, which is typical of plastic fracture (Figure 7b,e). In particular, the UT+SC alloy has a greater number of dimples that are deeper and smaller in size, which are uniformly distributed over the entire fracture surface, indicating superior ductility compared to the GC and SC alloys. Whether it is SC or UT+SC, squeeze casting has the potential to alter the fracture mechanism of alloy A from brittle to ductile fracture. Furthermore, it is evident from the illustrations in Figure 7e,f that there is no discernible compositional segregation, and the Cu-rich phase is uniformly dispersed in the UT+SC alloy, which indicates that UT can effectively improve the compositional segregation.

**Figure 8:**
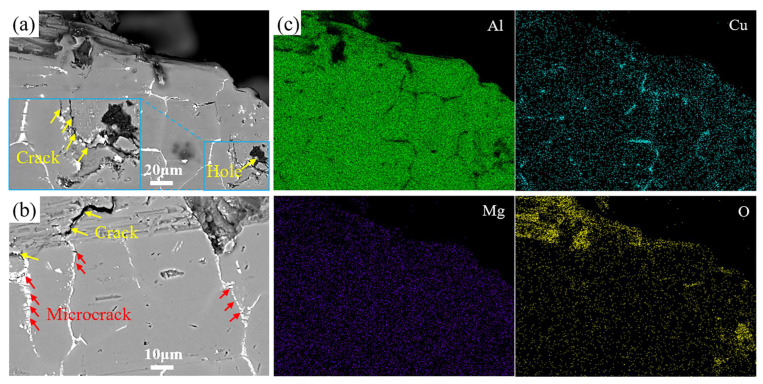
Microstructure along the lengthwise section of tensile specimens cut from GC (**a**) and UT+SC (**b**) alloys, and (**c**) the main elemental mapping distribution of the area in (**a**).

As we all know, ductility is significantly influenced by the generation and propagation behaviors of microcracks or cracks. In order to further clarify the evolution of the fracture behavior of different alloys, the fracture morphology along the longitudinal section of tensile specimens cut from the GC and UT+SC alloys, respectively, was assessed. Combined with Figure 8a and its elemental mapping distribution (Figure 8c), it can be clearly seen that the crack source of the GC alloy is the hole, which is due to the fact that pores readily induce stress concentration. Microcracks form the hole and continue to expand into cracks to cause fracture, which is one of the reasons for the poor mechanical properties of the GC alloy. Conversely, no pores were observed within the UT+SC alloy, and cracks originated from the second phase. Apparently, some microcracks extended along the second phase, but no obvious cracks were produced in the UT+SC alloy. Moreover, the second phase in the UT+SC alloy is also significantly refined; thus, an improvement in its ductility is conceivable. In conclusion, the problems of high porosity and severe compositional segregation in GC alloys are mitigated in SC and UT+SC alloys, which is a key contributor to the enhanced properties of SC and UT+SC alloys.

### 3.3. Effect of Sc Addition on the Microstructure and Properties of the Al-Li Cast Alloy

**Figure 9:**
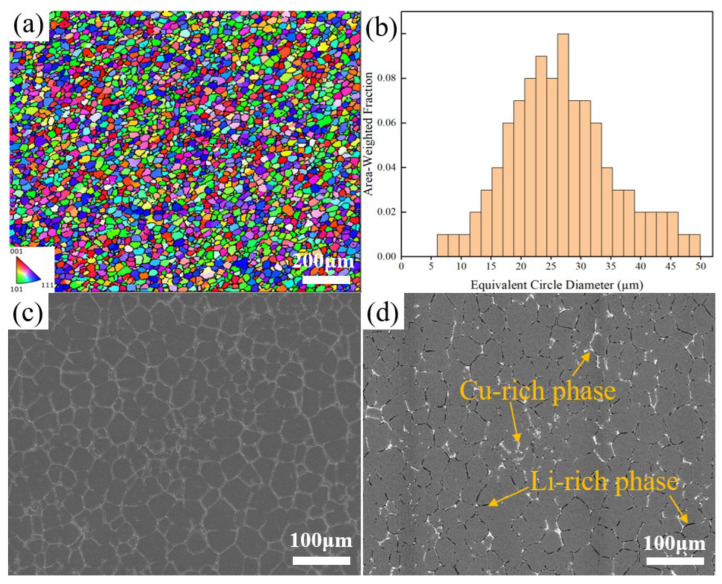
IPF with grain boundary (**a**). Grain size distribution (**b**). SEM (**c**) and BSE (**d**) images of alloy B.

**Figure 10:**
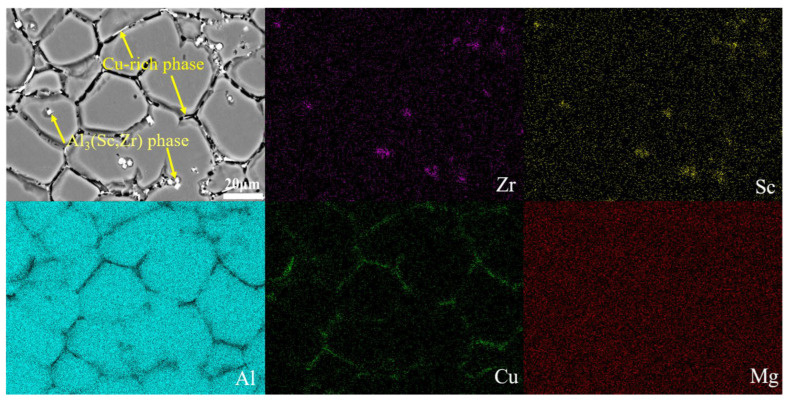
The elemental mapping distribution diagram of alloy B.

In addition to forming processes, microalloying is another effective method by which to improve the microstructure and enhance properties of Al-Li cast alloys. In the microalloying of this alloy, Sc is a critical element. From the IPF diagram of Al-2Li-2Cu-0.5Mg-0.2Zr-0.2Sc alloy (alloy B) in Figure 9a, it is observed that the addition of Sc results in a considerable reduction in grain size and enhanced grain roundness compared to the alloy without Sc (alloy A). According to the histogram of the EBSD equivalent circular diameter in Figure 9b, the grain size of alloy B is as small as 20 μm, which is 57% smaller than that of alloy A with a grain size of 44 μm. Alloy B is predominantly composed of the α-Al and second phases, including the Cu-rich phase and the Li-rich phase (Figure 9c,d). Notably, the Cu-rich phase exhibits a reticulated or semi-reticulated distribution. Similar to alloy A, as shown in Figure 4d, under the UT+SC process, the porosity and segregation of the composition of alloy B have been successfully eliminated. In addition, compared to alloy A, the dimension of the second phase in alloy B is significantly reduced under same process, while the second phase is more uniformly distributed. Based on the elemental mapping distribution of alloy B shown in Figure 10, except for the Cu-rich and Li-rich phases, a second phase containing Sc and Zr was found in some regions of alloy B. According to the Al-Sc binary phase diagram [20], when the cooling rate exceeds a certain threshold, non-equilibrium solidification occurs; i.e., an L→α-Al+Al_3_Sc eutectic reaction occurs. In accordance with the Al-Sc-Zr ternary phase diagram [21], Al_3_(Sc, Zr) phases may also be formed during the solidification process.

**Figure 11:**
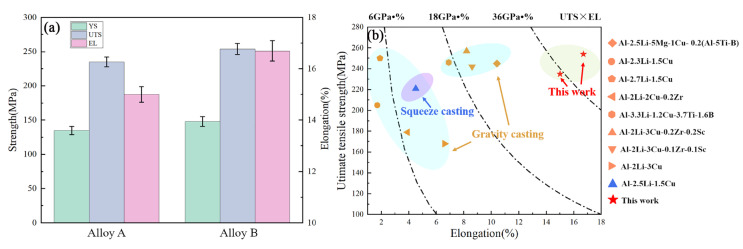
(**a**) As-cast tensile properties of two Al-Li alloys. (**b**) Comparison of properties with various Al-Li cast alloys prepared by other researchers using different casting methods [14,16,18,22,23].

To illustrate the enhancement of mechanical properties through the addition of Sc, a comparison between alloys A and B was conducted. As shown in Figure 11a, the UTS, YS, and elongation of alloy B are 254 MPa, 148 Mpa, and 16.7%, which are 8.1%, 9.6%, and 11.3% higher than those of alloy A, respectively. In order to highlight the advantages of the UT+SC technique developed in this study for the preparation of Al-Li cast alloys, a comparison of the properties of the two alloys (i.e., alloys A and B) with those of Al-Li cast alloys prepared by other researchers using different casting methods is shown in Figure 11b. The two Al-Li cast alloys prepared in this work have excellent ductility, reaching up to approximately 17%. Meanwhile, compared with other Al-Li cast alloys, the product of strength and elongation (i.e., UTS × EL) for alloy A reaches 36GPa•%, significantly surpassing those of other Al-Li cast alloys reported in the available literature. With the addition of Sc, the UTS × EL value of alloy B is further increased to 42GPa•%, which is 16.7% higher than that of alloy A. This confirms that the forming process innovation in this work can significantly improve the properties to a considerable extent, and also validates the feasibility of microalloying using Sc elements to improve the mechanical properties of the alloys.

## 4. Discussion

### 4.1. Mechanisms of Microstructure Evolution under Forming Processes and Sc Addition

**Figure 12:**
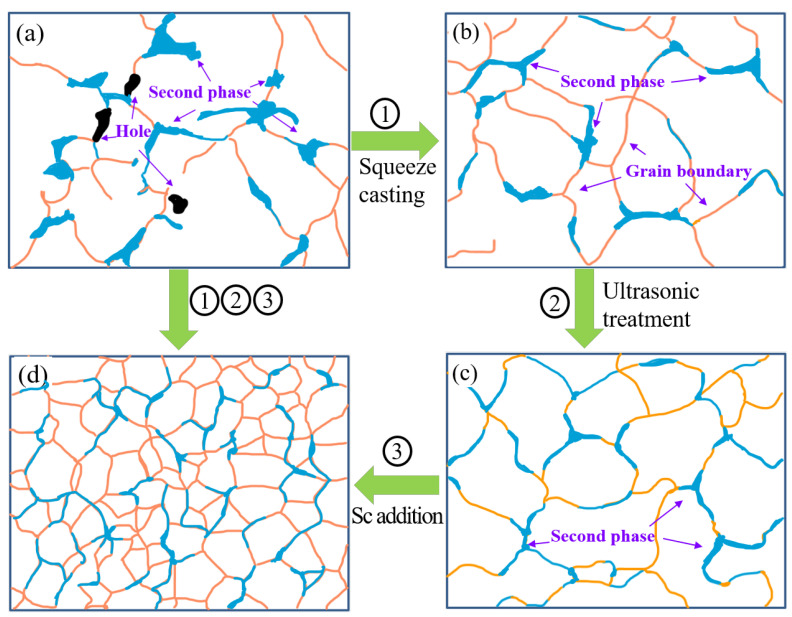
Schematic mechanism of the effect of forming process and Sc addition on the microstructure of Al-Li cast alloys: (**a**) microstructure sketch of alloy A (without Sc) by GC; (**b**) microstructure sketch of alloy A via SC; (**c**) microstructure sketch of alloy A via UT+SC; (**d**) microstructure sketch of alloy B (with Sc element) via UT+SC.

Figure 12 shows a schematic mechanism of the effect of the forming process and Sc addition on the microstructure of Al-Li alloys. From Figure 12a,b, compared to the GC alloy, the α-Al grains in the SC alloy are finer, and the size of the Cu-rich phases is also significantly refined, with the pores basically disappearing. This occurs because the high-pressure and rapid cooling conditions during squeeze casting facilitate non-equilibrium solidification of the melt, which reduces the atomic diffusion coefficient and consequently inhibits grain growth [24,25]. On the one hand, applying pressure can bring the melt and mold into close contact, increasing thermal conductivity and accelerating the cooling rate of the melt, which can refine the grains and crystalline phases and eliminate holes. On the other hand, the application of pressure alters the alloy’s phase diagram, resulting in a larger undercooling of the superheated alloy liquid, which improves the nucleation rate of solidification, thus refining the grains and crystalline phases [26].

Compared to the SC alloy, the α-Al grain size distribution in the UT+SC alloy is more uniform, as shown in Figure 12b,c. Furthermore, the segregation of Cu-rich phases basically disappears and eventually becomes homogeneous in the UT+SC alloy. This is mainly because high-energy ultrasonic waves in the melt will produce cavitation and acoustic streaming. First, the growth of cavitation bubbles induces significant heat absorption. Simultaneously, the high pressure resulting from micro-bubble collapse elevates the equilibrium solidification temperature of the microregional melt. This process results in localized supercooling of the metal melt, thereby enhancing nucleation [26,27]. Second, acoustic streaming can agitate the melt and inhibit dendrite growth, while the roots of the incipient dendrites will be fused in the shear, which further increases the nucleus count and refines the grains. It also provides a good degassing effect via acoustic streaming and cavitation, allowing the gas to be discharged [28]. The advantages of both processes (i.e., the rapid cooling conditions and high pressure of SC and the acoustic cavitation and streaming effects of UT) can be well amplified and superimposed, which is conducive to grain refinement, the reduction of solute element segregation, and the elimination of pores [29,30], thus further improving the plasticity and strength of SC alloy.

Moreover, the addition of Sc to Al-Li alloys is effective in terms of grain refinement. Figure 12c,d illustrate that the grain size is not only drastically reduced after Sc addition; the grain morphology is also refined from the dendritic crystals in the Sc-free alloy to the fine equiaxed structure. In addition, grain refinement is accompanied by a reduction in the second phase’s size and the uniform distribution of the second phase along the grain boundaries. In general, elemental Sc is divided into two parts, one in the form of solute atoms and the other in the incipient Al_3_(Sc, Zr) phase [31,32,33]. As mentioned above, adding Sc elements to the melt creates a large number of Al_3_Sc or Al_3_(Sc, Zr) particles. Al_3_Sc, or Al_3_(Sc, Zr), is an L1_2_-type ordered equilibrium phase, with a lattice constant of about 0.4103 nm and a density of about 3.026 g/cm^3^ [34]. Based on quantitative calculations, the mismatch between the Al_3_Sc or Al_3_(Sc, Zr) and the matrix is only 1.32%, and its precipitation temperature is higher than that of the α-Al phase. This suggests that Al_3_Sc or Al_3_(Sc, Zr) can effectively act as heterogeneous nucleation sites within Al alloys. Therefore, adding a small amount of Sc can have a significant grain refinement effect in the Al-Li cast alloy under the same forming process.

### 4.2. Mechanism for Change of Properties under Forming Processes and Sc Addition

The reduction in porosity, the uniform dispersion of secondary phases, and grain refinement contribute significantly to the enhanced strength and elongation of Al-Li alloys produced via UT+SC. Al-Li alloys typically absorb significant amounts of hydrogen during the melting and casting processes, which leads to porosity as a result of reduced solubility upon cooling. These pores act as stress concentration points and tend to promote the formation and expansion of cracks, ultimately reducing fatigue life and the tensile properties of the castings [35,36]. Reducing porosity in castings thus enhances the mechanical properties of the alloys. In the SC process, the high pressure can enhance the solubility of gas and the fluidity of the melt, effectively eliminating casting defects like shrinkage cavities and significantly improving the alloy’s strength and ductility. Refining the primary α-Al grains contributes to a reduction in the distance travelled by the solid–liquid solidification front as a result of the high solidification rate of the SC alloy. Grain refinement is also conducive to improving the distribution and morphology of the second phase while reducing compositional segregation, resulting in a significant improvement in the mechanical properties of the alloy [36].

Based on the Hall–Petch formula (δs=δ0+k⋅d−12), the smaller the grain size is, the greater the strength of the alloy becomes within a certain range of grain sizes [37], where $\sigma_{s}$ is the yield strength, d is the average grain diameter, and k is the Hall–Petch coefficient. For Al alloys, k = 0.04 MPa⋅m12 is generally considered [38], and the Hall–Petch coefficients for Al-Li alloys reported in some studies are 0.068~0.17 MPa⋅m12 [14,39]. According to the grain statistics, the GC, SC, and UT+SC alloys’ average grain sizes are about 186 μm, 104 μm, and 44 μm, respectively. Since the k value ranges from 0.04 to 0.23, the theoretical contribution of grain refinement to the strength of the SC alloy is about 1.0~5.7 MPa compared to the GC alloy. Compared to GC and SC alloys, the theoretical contribution of grain refinement to the strength of UT+SC alloys is about 3.1 to 17.8 MPa and 2.1 to 12.1 MPa, respectively. Compared to the GC alloy, the actual YS of the SC alloy is increased by 25 MPa, which is significantly greater than the maximum theoretical contribution of 5.7 MPa to strength from grain refinement; thus, the reduction in porosity is the main reason for the increase in the strength of the SC alloy. Compared to the SC alloy, the actual YS of the UT+SC alloy increased by 10 MPa, which is slightly lower than the maximum theoretical strength contribution of 12.1 MPa from grain refinement. This indicates that the increase in the strength of the UT+SC alloy is mainly due to the grain refinement and partially associated with the uniform distribution of the second phase. Therefore, SC and UT processes are critical for improving the microstructure and properties of Al-Li cast alloys.

The drastic grain refinement induced by Sc can effectively improve the mechanical properties of the Al-Li cast alloy [40,41,42]. As mentioned above, the average grain sizes of Sc-free and Sc-containing alloys are about 44 μm and 20 μm, respectively, with k values ranging from 0.04 to 0.23. Referring to the previous discussion on grain refinement strengthening, the theoretical contribution of grain refinement to the strength of alloy B ranges from about 2.9 to 16.8 MPa. In fact, the actual YS of the Al-Li cast alloy increases from 135 MPa to 148 MPa after Sc addition, with an increase of 13 MPa, which is slightly lower than the maximum strength contribution of 16.8 MPa provided by grain refinement. Therefore, the increase in mechanical properties with the addition of Sc results primarily from grain refinement and is partly attributable to other factors such as more grain boundaries and dispersed second phases.

## 5. Conclusions

The combination of UT (ultrasonic treatment) and SC (squeeze casting) processes has been proposed to prepare an Al-Li cast alloy for the first time, and the effects of forming processes and Sc addition on the microstructure and mechanical properties have been investigated. The findings can be summarized as follows:

1. Compared to the GC alloy, the SC alloy not only showed a 44.1% decrease in grain size but also a significant reduction in porosity, while compositional segregation still existed in both processes. After UT+SC, the α-Al grain of the Al-Li cast alloy was further refined from 104 μm to 44 μm, and its compositional segregation was effectively improved.
2. The ultimate tensile strength (UTS), yield strength (YS), and elongation of the UT+SC alloy reached 235 MPa, 135 Mpa, and 15%, respectively. These values were 113.6%, 28.6%, and 1150% higher than those of the GC alloy, while 6.8%, 8%, and 15.4% higher than those of the SC alloy, respectively. The combined effects of porosity reduction, grain refinement, and uniform distribution of second phases were mainly responsible for the significant increase in strength and elongation of the Al-Li cast alloy.
3. With the addition of 0.2 wt.% Sc element, the UT+SC alloy exhibited a better as-cast solidification microstructure, with the grain size reduced to as small as 20 μm, representing a 54.5% decrease compared to the Sc-free Al-Li cast alloy. The UTS, YS, and elongation of the Sc-containing Al-Li cast alloy are 254 MPa, 148 Mpa, and 16.7%, respectively, which are 8.1%, 9.6%, and 11.3% higher than the those of the alloy without Sc under the same UT+SC process. Interestingly, the product of strength and elongation (i.e., UTS × EL) for the two alloys reached 36 GPa•% and 42 GPa•%, respectively, which is much higher than that of the other Al-Li cast alloys reported in the available literature.

## Figures and Tables

**Table 1:** The actual chemical compositions of Al-Li cast alloys in this work (in wt. %).

Alloy	Li	Cu	Mg	Zr	Sc	Al
Alloy A	2.05	1.97	0.48	0.21	0	Bal.
Alloy B	1.98	2.03	0.51	0.19	0.19	Bal.

## Data Availability

The original contributions presented in the study are included in the article. Further inquiries can be directed to the corresponding author/s.
